# Plume Noise Suppression Algorithm for Missile-Borne Star Sensor Based on Star Point Shape and Angular Distance between Stars

**DOI:** 10.3390/s19183838

**Published:** 2019-09-05

**Authors:** Qiaoyun Fan, Zhixu Cai, Gangyi Wang

**Affiliations:** School of Instrumentation Science and Opto-electronics Engineering, Beihang University, Beijing 100191, China (Q.F.) (Z.C.)

**Keywords:** star sensor, plume noise, principal component analysis, shape features, angular distance

## Abstract

When a missile is launched, the plume generated by the propulsion system will produce a lot of fake stars in the star image, which will affect the normal work of the missile-borne star sensor. A plume noise suppression algorithm based on star point shape and angular distance between stars is proposed in this paper, which is a preprocessing algorithm for star identification. Firstly, principal component analysis is used to extract the shape features of star points. Secondly, the authenticity of star points is evaluated based on length-width ratios. Thirdly, in two consecutive frames of star images, according to the shape features of star points, the optimal matching window is determined to achieve accurate matching of the corresponding star points. Finally, the rapid elimination of fake stars is completed by the principle of invariant angular distance between true stars. Simulation experiment results show that the proposed algorithm is quite robust and fast, and the elimination ratio is high even if the number of fake stars reaches four times more than true stars. Compared with the existing star identification algorithms, when the number of fake stars is large, the advantage of the proposed algorithm is obvious. Experimentation on actual star images verifies that the proposed algorithm can meet the requirements of spacecraft even if there are a large number of fake stars in the star image.

## 1. Introduction

The star sensor is an important kind of attitude measurement instrument which is widely used in satellites and missiles with advantages of high precision and no drift. The working process of the missile-borne star sensor includes four steps: Star image acquisition, star point centroid extraction, star identification, and attitude calculation.

During the missile launching process, the plume generated by the propulsion system combustion is micro-manifested as a large number of dust particles, which scatter sunlight or other stray light to form luminous bodies, thus forming fake star points in the star image. These fake star points cannot be distinguished from true star points in brightness, which have a serious influence on star centroid extraction, resulting in star identification and attitude calculation taking a long time and even causing errors.

As there are few studies on how to suppress plume noise at present, the mainstream solution is to adopt star identification algorithm which is more robust to fake stars. The grid algorithm and its subsequent improved algorithms (Padgett and Kreutz-Delgado, 1997 [1]; Lee and Bang, 2007 [2]; Na et al., 2009 [3]) are robust to position noise and fake stars, and fast, but require a sufficient number of true stars to be effective. The triangle algorithm [4] is the most widely used star identification algorithm, which uses three stars and their angular distances to generate a triangle feature. The algorithm is effective even if there are only three stars in the star image. Mortari et al. proposed the pyramid algorithm [5] which is quite robust to fake stars, but with the number of fake stars increasing, the algorithm becomes very slow. Kolomenkin et al. proposed a geometric voting algorithm [6], which uses a voting method to find the relationship between image stars and catalog stars. However, when the number of fake stars is large, the efficiency and accuracy of the algorithm decrease significantly. Delabie et al. proposed an algorithm based on the shortest distance transform [7]. The algorithm is robust to fake stars, but when some true stars are missing, the identification ratio will decrease, and the time efficiency of the algorithm is low. Zhao et al. proposed an algorithm based on K–L transformation and star walk formation [8], which is fast and consumes little memory, but is not quite robust to fake stars.

Li et al. proposed a novel guide star catalog generation algorithm [9], which is not a star identification algorithm, but provides reliable and efficient performance for the star sensor. Some algorithms [10,11] use multi-frames processing to improve robustness, but the identification time cannot meet the real-time requirements. Vincenzo Schiattarella et al. proposed a multi-poles algorithm [12], which is quite robust to spurious targets but has poor dynamic performance. The performance of this algorithm decreases with increasing angular velocity of the star sensor. Wang et al. proposed a star identification algorithm based on hash map [13], which maps each triangle feature to an integer and builds a hash map of all the triangle features. This algorithm is quite robust to fake stars, and its identification ratio is similar to the pyramid algorithm, while its speed is much faster than that of the pyramid algorithm. For high dynamic star points, Sun et al. proposed a smearing model under conditions of variable angular velocity [14] and a motion-blurred star acquisition method [15]. Fan et al. proposed a voting-based star identification algorithm utilizing local and global distribution [16], which is fast and needs less memory, but it is not robust enough to fake stars. Wang et al. proposed a false star filtering algorithm for star sensor based on angular distance tracking [17], which can filter out a large amount of fake stars but needs a long time.

Although fake star points cannot be distinguished from true star points in brightness, the plume is generated by the propulsion system, whose moving speed is much faster than true stars. Therefore, the shape is different for fake star points and true star points. Taking full advantage of shape features and combining the angular distance between stars, we propose an effective missile plume noise suppression algorithm for missile-borne star sensor.

The remainder of this paper is organized as follows: In Section 2, the principles and details of the proposed algorithm are described; in Section 3, performance of the proposed algorithm is evaluated on both simulation star images and actual star images; and in Section 4, conclusions of the proposed algorithm are drawn.

## 2. Method Description

In this paper, a star point is regarded as an ellipse, and the ratio of its long side to short side is defined as the length-width ratio. In general applications, true star points approximately conform to 2-D Gaussian distribution, the length-width ratio of which is small, while fake star points approximately conform to long strip distribution, the length-width ratio of which is large. In a few cases where there is little difference in length-width ratio between true star points and fake star points, they can be distinguished by the angular distance between stars.

### 2.1. Star Shape Features Extraction Based on PCA

**Figure 1:**
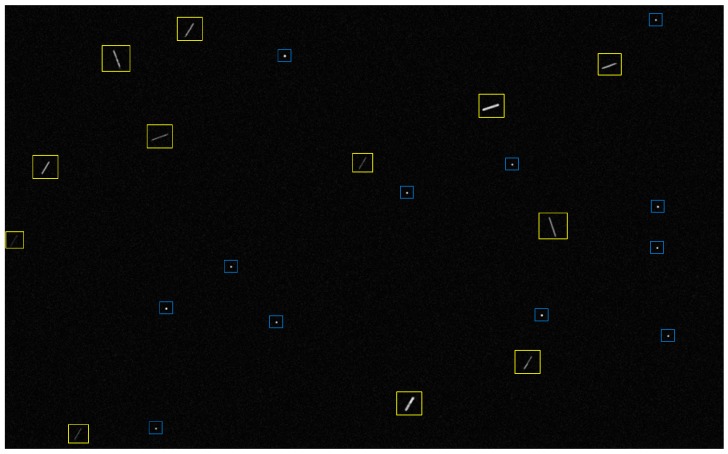
Star image containing fake star points.

In Figure 1, true star points are in blue windows, and fake star points are in yellow windows. Due to the irregular shape of particles and the complexity of reflecting light sources, fake star points are uneven in gray distribution, poor in continuity, and random in movement direction.

Principal component analysis (PCA), also known as eigenvector transformation, aims to transform a given set of dependent variables into another set of independent variables through dimensionality reduction. The new set of variables are arranged according to the variance from large to small. The largest variance is the first principal component, and the second largest variance is the second principal component, and so on. PCA is widely used in data compression, image rotation, feature selection, and statistical recognition of remote sensing multispectral images.

The shape features of a star point include moving direction, length, width, and length-width ratio. A star point is composed of a series of discrete pixels, and each pixel is a two-dimensional vector, including abscissa and ordinate. PCA is used to process the star point pixels, which can quickly and accurately calculate the shape features [18]. The moving direction of star point is the principal component direction, and the degree of data dispersion is large. While in the direction perpendicular to the moving direction, the degree of data dispersion is small.

Suppose a star point contains *N* pixels with coordinates of $\left(x_{i},y_{i})\;(i=1,2,\cdots ,N\right)$, and each pixel is considered as a two-dimensional vector:

(1) $$v_{i}=\left[\begin{array}{l} x_{i}\\ y_{i} \end{array}\right]$$

The mean of these vectors is

(2) $$m=E\left\{v\right\}=\frac{1}{N}\left[\begin{array}{l} \sum_{i=1}^{N}x_{i}\\ \sum_{i=1}^{N}y_{i} \end{array}\right]$$

The feature covariance matrix is

(3) $$H=E\left\{(v-m){\left(v-m\right)}^{T}\right\}=\frac{1}{N}\sum_{i=1}^{N}v_{i}v_{i}^{T}-mm^{T}$$

The diagonal of *H* includes the variance of *x* and *y*, and the non-diagonal includes the covariance of *x* and *y*. Since the pixel coordinates are all real numbers, *H* is a 2 × 2 real symmetric square matrix. Therefore, there must be a unit orthogonal matrix $P=(p_{1},p_{2})$:

(4) $$P^{-1}HP=\left[\begin{array}{cc} \lambda_{1} & 0\\ 0 & \lambda_{2} \end{array}\right]$$

where $\lambda_{1},\lambda_{2}$ are the eigenvalues of matrix *H*, respectively describing the size of the eigenvectors $p_{1},p_{2}$, and $p_{1},p_{2}$ describe the direction of the star points. Suppose $\lambda_{1}\geq \lambda_{2}$, $p_{1}$ is the principal component direction, which is the moving direction of the star point, and $p_{2}$ is its vertical direction.

**Figure 2:**
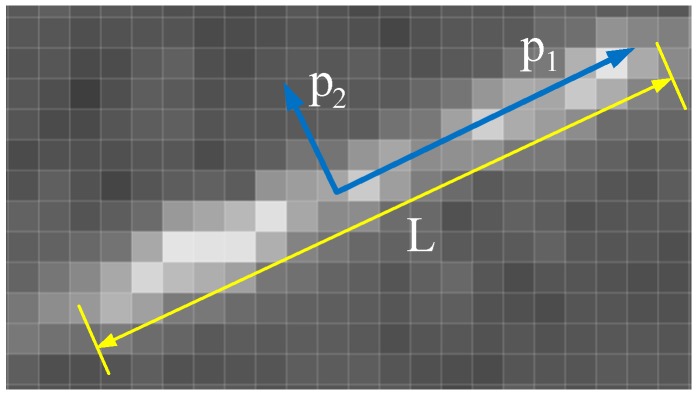
Shape features of a star point.

As shown in Figure 2, according to the eigenvalues $\lambda_{1},\lambda_{2}$ and the number of pixels *N*, the shape features of the star point can be calculated:

(5) $$r=\frac{\lambda_{1}}{\lambda_{2}}$$

(6) $$L=\sqrt{Nr}$$

where *r* is the length-width ratio of the star point, *L* is the length of the circumscribed rectangle of the star point, and the eigenvector $p_{1}$ is the moving direction of the star point.

### 2.2. Evaluate the Authenticity of Star Points Based on Length-Width Ratios

**Figure 3:**
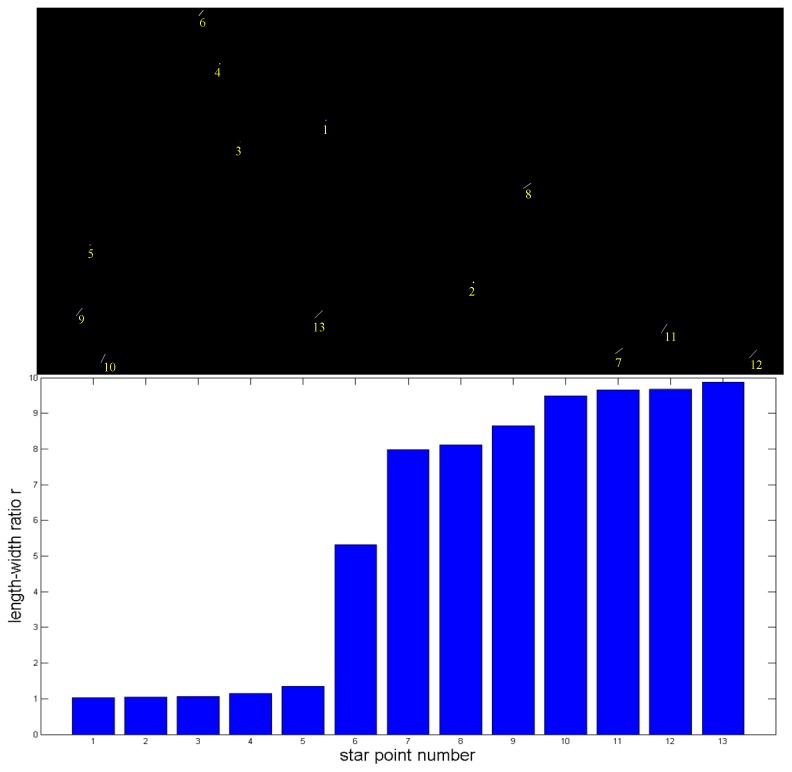
Sort length-width ratios of star points.

In general applications, true star points approximately conform to 2-D Gaussian distribution, the length-width ratio of which is small. While fake star points approximately conform to long strip distribution, the length-width ratio of which is large. Sort the *N* star points in an image from small to large according to the length-width ratio $r_{i}$, as shown in Figure 3.

Calculate $\sigma_{i,i-1}$ between adjacent length-width ratios:

(7) $$\sigma_{i,i-1}=\frac{r_{i}}{r_{i-1}}(r_{i}-r_{i-1})\;(i=2,\cdots ,n,\;\mathrm{set}:\;\sigma_{1,0}=0)$$

In this paper, the authenticity of star points is evaluated according to the length-width ratio *r_i_*. When $r_{i}$ is smaller, the authenticity of the star point is higher. When $r_{i}$ is larger, the authenticity of the star point is lower. $\sigma_{i,i-1}$ describes the change range of $r_{i}$. When $\sigma_{k,k-1}=\max(\sigma_{i,i-1})$, it indicates that the length-width ratio has changed significantly. Therefore, $r_{k-1}$ is judged to be the largest length-width ratio of star points with high authenticity, while $r_{k}$ is judged to be the smallest length-width ratio of star points with low authenticity.

According to $\sigma_{k,k-1}$ and $r_{k}$, the star points in the image can be classified. For star points whose length-width ratio is greater than $2r_{k}$, it means that the length-width ratio is too large, and these star points are classified as $C_{3}$ which are judged to be fake stars and eliminated. For star points whose length-width ratio is smaller than $r_{k}$, they are classified as $C_{1}$ which has high authenticity. For star points whose length-width ratio is between $r_{k}$ and $2r_{k}$, they are classified as $C_{2}$ which has low authenticity.

(8) $$\mathrm{star}\;\mathrm{points}\in \left\{ \begin{array}{ll} C_{1}=\{r_{1},r_{2},\cdots ,r_{k-1}\}, & k=\{i|\sigma_{k,k-1}=\max(\sigma_{i,i-1})\}\\ C_{2}=\{r_{k},r_{k+1},\cdots ,r_{m-1}\}, & r_{m-1}\leq 2r_{k}\\ C_{3}=\{r_{m},r_{m+1},\cdots ,r_{N}\}, & r_{m}>2r_{k} \end{array}\right.$$

For the two classes $C_{1}$ and $C_{2}$, this paper will further distinguish according to the angular distance between stars. In two consecutive frames of star images, the angle between any two true star vectors is constant, while the angle between fake stars is probable not. Based on this rule, the elimination of fake star points can be performed.

### 2.3. Accurate Matching of Corresponding Star Points

Matching the corresponding star points involves finding the position of the same star point in two frames. By superimposing two consecutive frames of star images, take the star point belonging to the second frame as the original star point. Around the original star point, find the matching star point belonging to the first frame. The size of the matching window is an important parameter—a large window will contain redundant star points while a small window will miss the matching star point. In this paper, based on the length of the original star point, the optimal matching window is determined to achieve accurate matching of the corresponding star points.

**Figure 4:**
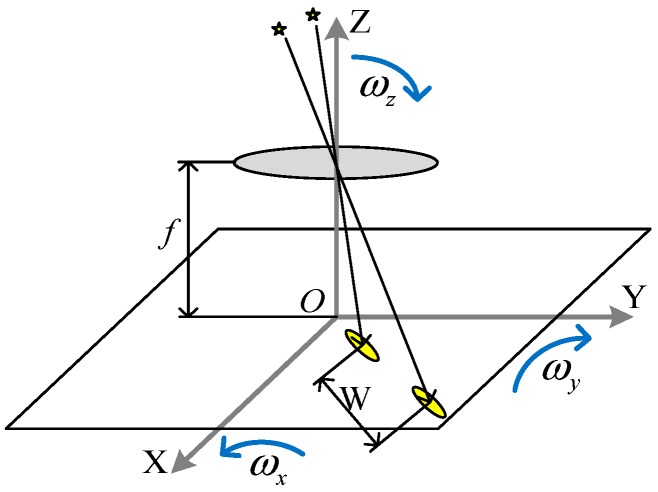
Calculating principle of matching window.

As shown in Figure 4, *W* is the distance between the centroids of the corresponding star points. $\omega_{x},\omega_{y},\mathrm{and}\;\omega_{z}$ are the three-axis angular velocity of the star sensor. *f* is focal length of the lens. *D* is pixel size of the image sensor. *T* is exposure time of the image sensor. *t* is the time between the end of first frame exposure and the beginning of second frame exposure.

When length-width ratio *r* is large, the star point is of long strip distribution in shape. In this case, the velocity of the star point on image is L/T, where *L* is the length of the star point. *W* is calculated as

(9) $$W=\frac{L}{T}\cdot (T+t)$$

When the length–width ratio *r* is small, the star point is of 2-D Gaussian distribution in shape, so the error of L/T becomes large. In this case, the probability of the star point being a true star is high, and the velocity of the star point on image is mainly determined by $\omega_{x},\omega_{y},\mathrm{and}\;\omega_{z}$. The centroids of the star points $\left(x_{0},y_{0}\right)$ and $\left(x_{T+t},y_{T+t}\right)$ satisfy

(10) $$\left(\begin{array}{c} x_{T+t}\\ y_{T+t}\\ -L_{f} \end{array}\right)=A^{T+t}\cdot \left(\begin{array}{c} x_{0}\\ y_{0}\\ -L_{f} \end{array}\right)$$

where $L_{f}=f/D$. The attitude transformation matrix $A^{T+t}$ is

(11) $$A^{T+t}=I-\omega (T+t)=\left[\begin{array}{ccc} 1 & \omega_{z}(T+t) & -\omega_{y}(T+t)\\ -\omega_{z}(T+t) & 1 & \omega_{x}(T+t)\\ \omega_{y}(T+t) & -\omega_{x}(T+t) & 1 \end{array}\right]$$

$\left(x_{T+t},y_{T+t}\right)$ is calculated as

(12) $$\left\{ \begin{array}{l} x_{T+t}=\frac{x_{0}+y_{0}\omega_{z}(T+t)+L_{f}\omega_{y}(T+t)}{\left[-x_{0}\omega_{y}(T+t)+y_{0}\omega_{x}(T+t)\right]/L_{f}+1}\\ y_{T+t}=\frac{y_{0}-x_{0}\omega_{z}(T+t)-L_{f}\omega_{x}(T+t)}{\left[-x_{0}\omega_{y}(T+t)+y_{0}\omega_{x}(T+t)\right]/L_{f}+1} \end{array}\right.$$

According to the parameters of the star sensor in this paper, $\left|-x_{0}\omega_{y}(T+t)+y_{0}\omega_{x}(T+t)\right|\ll L_{f}$, Equation (12) can be simplified as

(13) $$\left\{ \begin{array}{l} x_{T+t}=x_{0}+y_{0}\omega_{z}(T+t)+L_{f}\omega_{y}(T+t)\\ y_{T+t}=y_{0}-x_{0}\omega_{z}(T+t)-L_{f}\omega_{x}(T+t) \end{array}\right.$$

In this case, W is calculated as

(14) $$W=\sqrt{{\left(y_{0}\omega_{z}+L_{f}\omega_{y}\right)}^{2}+{\left(x_{0}\omega_{z}+L_{f}\omega_{x}\right)}^{2}}\cdot (T+t)$$

In this paper, according to the parameters of the star sensor and experimental result, r=1.6 is taken as the threshold to judge whether a star point is of long strip distribution. For r>1.6, take 1.2 times of the calculated W to ensure the allowance. For r≤1.6, set $\omega_{x}=\omega_{y}=\omega_{z}=0.5{}^{\circ}/s$, so that W can contain all the star points of Gaussian distribution. Therefore, W is calculated as

(15) $$W=\left\{ \begin{array}{ll} \frac{L}{T}(T+t) & r>1.6\\ \sqrt{{\left(y_{0}\omega_{z}+L_{f}\omega_{y}\right)}^{2}+{\left(x_{0}\omega_{z}+L_{f}\omega_{x}\right)}^{2}}\cdot (T+t) & r\leq 1.6 \end{array}\right.$$

**Figure 5:**
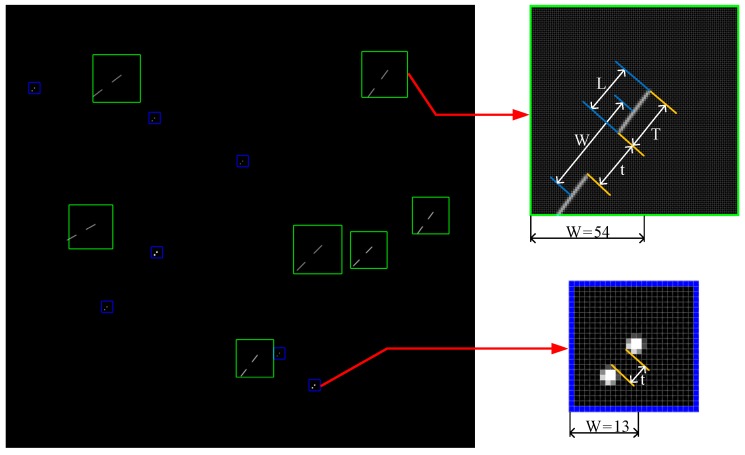
Star images matching.

When the window size W is large, it may contain other star points. For this case, it will be processed in the elimination of fake stars. As shown in Figure 5, two star images are matched according to the above algorithm, where the Gaussian distribution star points are located in blue matching windows, and the long strip distribution star points are located in green matching windows.

### 2.4. Rapid Elimination of Fake Stars

Most existing star identification algorithms construct features in a single frame through angular distance between stars or geometric distribution of stars, then find corresponding matching stars in the navigation star database. The amount of navigation star database is huge. When the number of fake star points is too large, the matching search time will increase non-linearly and sharply, and a large number of mismatches will occur.

In two consecutive frames of star images, the angle between any two true star vectors is constant, while the angle between fake stars is probable not because of their fast and irregular movement. Based on this principle, combined with the evaluation of the authenticity of star points and the accurate matching of corresponding star points in two frames, the final elimination of fake stars can be completed rapidly.

**Figure 6:**
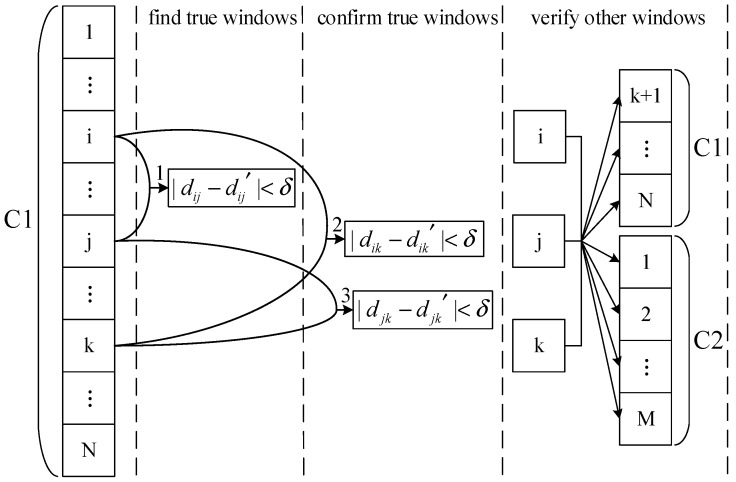
The flow diagram of eliminating fake stars.

Suppose the angular distance between the two star points in the first frame is *d*, and the angular distance between them in the second frame is $d^{\prime }$. If $|d-d^{\prime }|<\delta$ (in this paper, δ=0.002), the two star points satisfy the principle of invariant angular distance. As shown in Figure 6, the elimination algorithm includes the following three steps:

(1) Find True Star Windows:

The star points have been sorted according to the authenticity in $C_{1}$ and $C_{2}$. Therefore, from i=1,2,⋯,N−1 and j=i+1,⋯,N, verify whether i,j satisfy the invariant angular distance. If they satisfy, go to step (2).

(2) Confirm True Star Windows:

Although the star windows i,j satisfy the invariant angular distance, they need further verification by the triangle rule. That is, from k=j+1,⋯,N, confirm whether i,k and j,k both satisfy the invariant angular distance. If i,j,k satisfy the triangle rule, it indicates they are true star windows. If not until k=N, return step (1) and execute from j+1.

(3) Verify Other Windows:

Based on i,j,k, the remaining windows of $C_{1}$ and all windows of $C_{2}$ are verified by the triangle rule. If the verified window satisfies the invariant angular distance, it is a true star window, otherwise it is a fake star window.

In step (1) to step (3), if there are two or more matching star points in the window, the algorithm will first use the star point whose length is close to the original star point. If this star point does not satisfy the invariant angular distance, the algorithm will use another matching star point.

In step (1) and step (2), if there are no i,j,k that satisfy the invariant angular distance in $C_{1}$, the algorithm will add $C_{2}$ to the matching windows queue.

## 3. Experimental Results and Discussion

### 3.1. Simulation Experiment

#### 3.1.1. Parameter Selection

We developed a simulation program which can generate star images with different number of fake stars, and with variable position noise and magnitude noise. Table 1 lists the key parameters:

In the simulation experiment, the exposure time of the simulation program is set to 100 ms. The standard deviation of position noise is set to 1.0 pixel, and the standard deviation of magnitude noise is set to 0.5 Mv. The number of fake stars is 20%, 40%, 60%, 80%, 100%, 120%, 140%, 160%, 180%, 200%, 220%, 240%, 260%, 280%, 300%, 320%, 340%, 360%, 380%, and 400% of the number of true stars, and 10,000 groups (each group contains two frames) of star images with random angle are generated in each case. The experiment is carried out on Windows 10, Core i3-8100@3.6G, and MATLAB R2016a.

The parameters of the proposed algorithm are listed in Table 2.

#### 3.1.2. Fake Star Elimination Experiment

**Figure 7:**
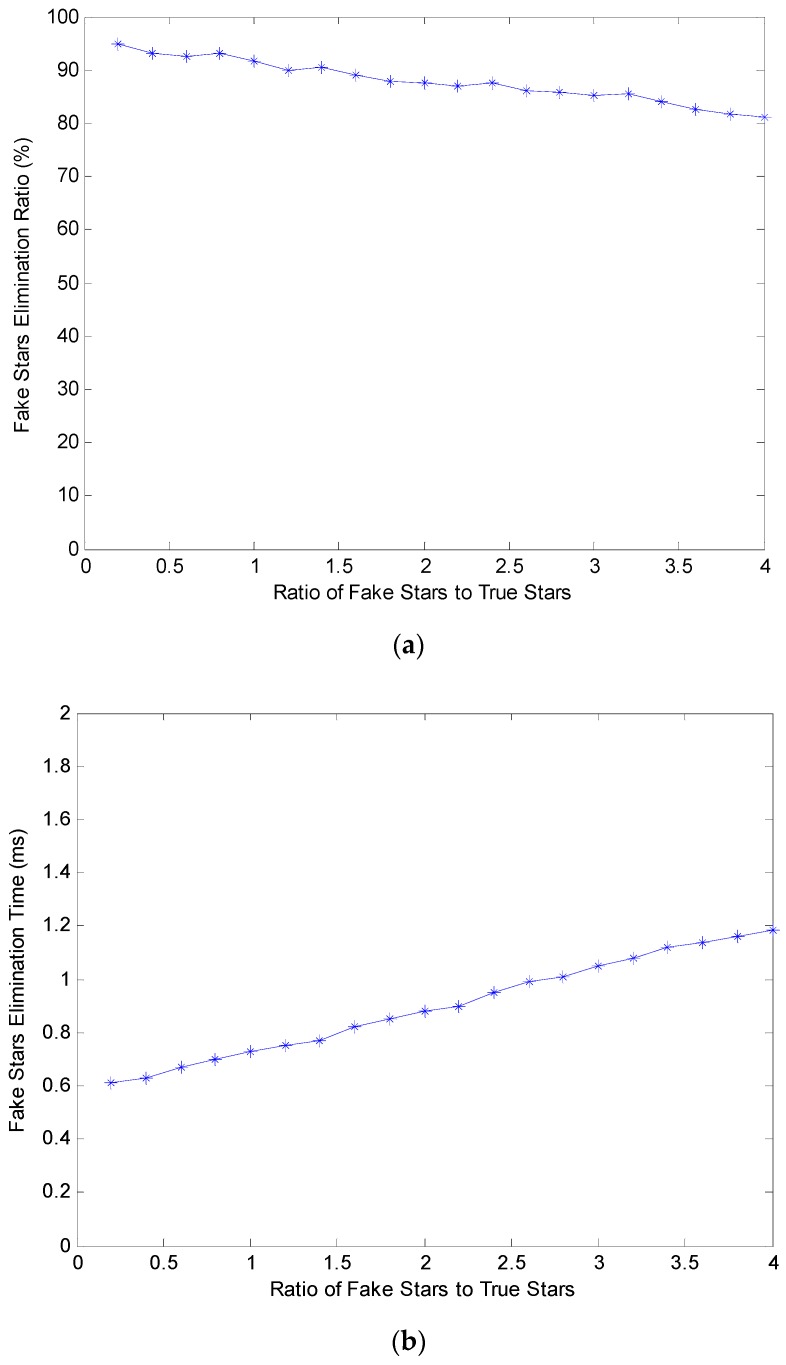
(**a**) Elimination ratio; (**b**) Elimination time.

The experiment is carried out on 20 × 10,000 groups of simulated star images, and the elimination ratio and elimination time of each group are determined. In this experiment, the elimination ratio is the percentage of the eliminated fake stars to all fake stars. The elimination time includes evaluating the authenticity of star points, matching corresponding star points, and eliminating fake stars. The experimental results are shown in Figure 7.

As shown in Figure 7, the fake star elimination ratio of the proposed algorithm is quite high, and the elimination time increases slowly with increasing number of fake stars. Even if the number of fake stars reaches four times that of true stars, the elimination ratio is still more than 80% and the elimination time is only 1.2 ms.

#### 3.1.3. Contrast Experiment

In order to better analyze the characteristics of the proposed algorithm, the hash map algorithm [13] and the pyramid algorithm [5] were adopted as references. As the proposed algorithm is a preprocessing algorithm for star identification, we chose the pyramid algorithm for subsequent star identification. Then it can be compared with the hash map algorithm and the pyramid algorithm. The later experiments are all in this way.

**Figure 8:**
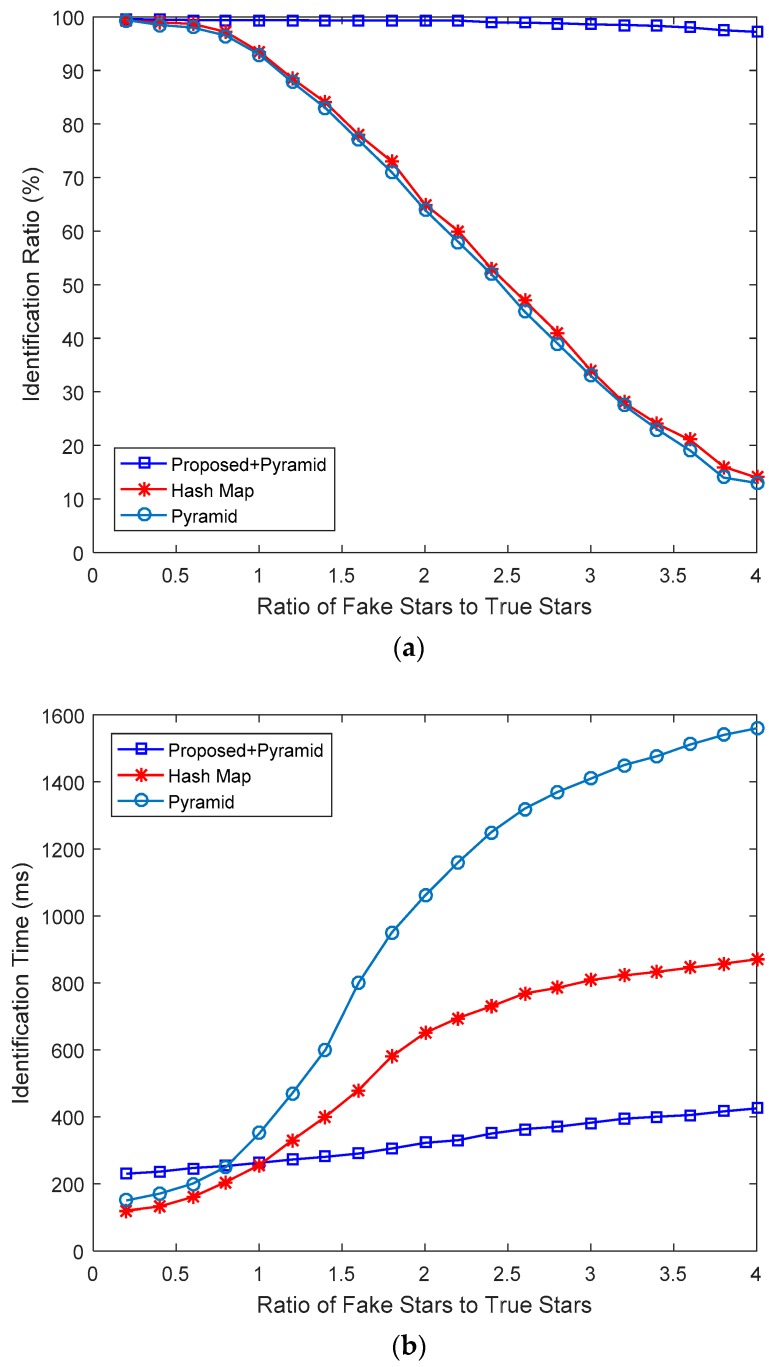
(**a**) Identification ratio; (**b**) Identification time.

The experiment is carried out on 20 × 10,000 groups of simulated star images, and the identification ratio and the identification time of the proposed algorithm with the hash map algorithm and the pyramid algorithm were compared. In this experiment, the identification ratio is the ratio of getting the correct attitude, and the identification time is the time consumed from the exposure of the star sensor to the calculation of attitude. The experimental results are shown in Figure 8.

As shown in Figure 8, with the number of fake stars increasing, the identification ratio of the hash map algorithm and the pyramid algorithm decreases significantly, while the proposed algorithm is almost unaffected. Even if the number of fake stars reaches four times that of true stars, the identification ratio of the proposed algorithm can still reach more than 96%. Since the proposed algorithm needs two consecutive frames of star images, the identification time is longer than that of the other two algorithms when the number of fake stars is small. However, with the number of fake stars increasing, the identification time of the proposed algorithm is much lower than that of the hash map algorithm and the pyramid algorithm.

### 3.2. Experiment on Actual Star Images

The parameters of the proposed algorithm are the same as those in Table 2. The parameters of the hash map algorithm are the same as those in [13]. The parameters of the pyramid algorithm are the same as those in [5]. The parameters of the star sensor are the same as those in Table 1. The CPU running the algorithms is an ARM processor.

Since the missile is too fast, there is no time to transmit the plume star images to the ground. In order to fully test the proposed algorithm, several actual star images obtained at different angular velocity are superimposed according to random position. In this way, an actual plume image can be simulated more practically and sufficiently.

At the Xinglong Astronomical Observatory of Chinese Academy of Sciences, the star sensor is mounted on the three-axis turntable, and several groups of star images are taken at different angular velocities. Table 3 is an example.

**Figure 9:**
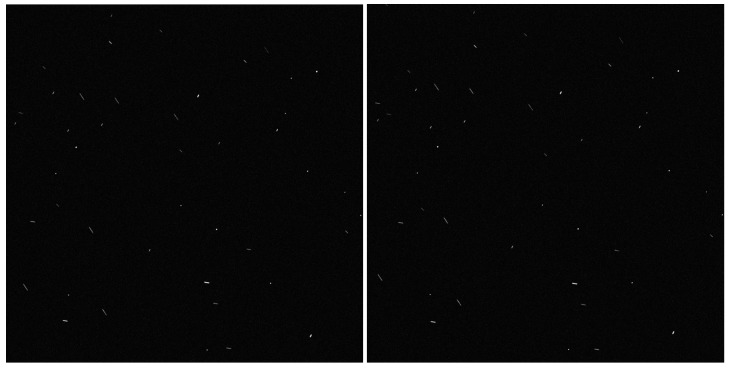
Generated actual plume star image.

According to Table 3, star image 1 is taken as the datum, the star points in images 2–5 are extracted, and these star points are superimposed into star image 1 according to random position as fake star points caused by the plume, thereby generating a plume star image as shown in Figure 9.

Based on the above method, 100 groups of star images are generated, and these images are read into the chips of the star sensor which is separately running the proposed program, the hash map program, and the pyramid program. The average processing time and the identification ratio in each case are compared as shown in Table 4.

As shown in Table 4, the identification ratio of the hash map algorithm is 67%, which shows that it identified 67 images correctly in total. The identification ratio of the pyramid algorithm is only 65%, and its average processing time is too long. The average processing time of the proposed algorithm is 112 ms and the identification ratio is 96%, which is much better than the other two algorithms.

## 4. Conclusions

A plume noise suppression algorithm based on star point shape and angular distance between stars is proposed in this paper. The proposed algorithm needs two consecutive frames of star images, and extracts the shape features of star points by principal component analysis. Then the authenticity of star points is evaluated based on length–width ratios, and accurate matching of corresponding star points is completed. Finally, the fake star points are eliminated rapidly based on the principle of invariant angular distance between true stars.

Experimental results on both simulation images and actual star images show that, when there is a large amount of fake star points in the image, the star sensor based on the proposed algorithm is not only robust, but also fast in computation. Therefore, the proposed algorithm has more advantages for missile navigation guidance.

## Figures and Tables

**Table 1:** Parameters of the Simulation Program.

Parameter	Value
Field of view	∅10°
Pixel size	0.0055 mm
Focal length	64.374 mm
Resolution	2048 × 2048 pixels
Max magnitude	5.5

**Table 2:** Parameters of the proposed algorithm.

Parameter	Value
Threshold *r* in Equation (15)	*r* = 1.6
Angular velocity in Equation (15)	$\omega_{x}=\omega_{y}=\omega_{z}=0.5{}^{\circ}/s$
Threshold to judge invariant angular distance	δ=0.002

**Table 3:** Star images at different angular velocity.

	1	2	3	4	5
$\omega_{x}({}^{\circ}/s)$	0.05	0.6	0.2	−0.7	1.8
$\omega_{y}({}^{\circ}/s)$	0.05	0.3	1.3	0.7	−1.2
$\omega_{z}({}^{\circ}/s)$	0	0	0	0	0

**Table 4:** The experimental result of generated plume images.

	Average Processing Time	Identification Ratio
**Proposed + Pyramid**	112 ms	96%
**Hash Map**	106 ms	67%
**Pyramid**	223 ms	65%
